# The impacts of development finance for climate on public health outcomes and life satisfaction: evidence from developing countries in the global south

**DOI:** 10.3389/fpubh.2026.1775273

**Published:** 2026-04-29

**Authors:** Dinkneh Gebre Borojo, Jiahui Liu

**Affiliations:** 1 School of Modern Business, Yiyang Vocational and Technical College, Yiyang, China; 2 School of Economics and Management, Yiyang Vocational and Technical College, Yiyang, China

**Keywords:** crude death rate, developing countries, development finance for climate, global south, IV-GMM, life satisfaction, public health

## Abstract

This study assesses how development finance for climate (DFC) affects public health outcomes and life satisfaction in developing countries across the global south (GS), using panel data for 69 developing countries from 2011 to 2021. The instrumental-variable generalized method of moments (IV-GMM) is used to address endogeneity. Several alternative approaches are used for robustness analysis. The findings provide robust evidence that increased DFC is positively associated with improvements in public health outcomes and life satisfaction in developing countries in the GS. More specifically, a 1% increase in DFC is associated with a 0.025% decrease in the crude death rate and a 0.047-point increase in the life satisfaction score, suggesting that increasing DFC to promote climate resilience and mitigation yields broader wellbeing gains beyond environmental benefits for developing countries in the GS. Moreover, heterogeneity analyses indicate that the positive effects of DFC on public health and life satisfaction are more pronounced among developing countries with low health expenditure and greater exposure to environmental risks. Additionally, the effects of DFC on public health and life satisfaction vary across income groups: middle-income countries are better able to leverage DFC to improve public health outcomes and life satisfaction than low-income developing countries. Finally, crude death rate has a significant and partial mediation role in the link between DFC and life satisfaction. Therefore, the results provide multifaceted evidence of DFC’s effects on the health and social wellbeing of developing countries in the GS and offer important policy recommendations aligned with SDG-3.

## 1 Introduction

Climate change is a major global health threat today (1). It leads to increasingly frequent and intense natural hazards, including extreme storms, flooding, and prolonged droughts (2). These events have both direct and indirect health effects, including increased mortality, spread of infectious diseases, and the triggering of health disasters (3). Also, climate change increases the likelihood of deadly disease outbreaks and pandemics, worsens maternal and child health outcomes, and amplifies the health impacts of extreme weather events (1). Besides, it harms ecosystems that support human life, ultimately endangering health (4). It places strain on health systems by negatively affecting healthcare workers and infrastructure, thereby hindering progress toward universal health coverage (5).

The unprecedented impact of climate change is disproportionately burdening developing countries in the GS, even though they contribute little to global greenhouse gas emissions (1). They are severely affected by climate change due to their high vulnerability, widespread poverty, low adaptive capacity, and limited resources to mitigate its impacts, particularly on health (1). Therefore, they face heightened exposure to severe climate shocks, including droughts, flooding, and cyclones. As a result, shortage of clean and safe water, lack of adequate food and clean air, and biodiversity loss due to climate change effects are critical factors adversely affecting human health by increasing the incidence of water-borne illnesses, malnutrition, injuries, mortality, heat-related stress, cardio-respiratory conditions, and other health challenges in developing countries (6). Thus, inadequate health outcomes adversely affect overall societal wellbeing (7).

Public health outcomes, represented by the crude death rate, are a direct indicator of a population’s health status (8) and reflect the overall likelihood of death within a population over a given period, helping to evaluate national health conditions. Therefore, it is an important indicator for evaluating and comparing public health outcomes across countries (9). Moreover, life satisfaction measures approximate the notion of utility or subjective wellbeing and represent a condition characterized by good health, happiness, and economic prosperity (10). It measures individuals’ subjective wellbeing within society (11). A key research gap concerns how developing countries in the GS can enhance public health outcomes and life satisfaction while addressing the health impacts of climate change. To curb these adverse effects, a range of climate-related policies has been implemented at both national and international levels. Within this framework, development finance for climate (DFC) has emerged as a critical policy instrument, intended to bridge financing gaps and support climate response efforts. Importantly, DFC represents an additional flow of resources from developed economies to enable developing countries to mitigate and adapt to climate change (12).

In practice, the connection between DFC and public health outcomes and life satisfaction rests on the idea that policy actions aimed at improving environmental performance, enhancing public health, and increasing life satisfaction are interconnected. For example, climate-related finance, intended to improve environmental performance, will also boost social wellbeing (11). It is also argued that maintaining and identifying additional sources of finance are essential for the functioning and strengthening of social protection systems in a changing climate. Moreover, social protection has gained prominence within the UN climate framework, reflected in its inclusion in decisions at COP28. Promoting sustainable development and wellbeing remains central to environmental governance and multilateral agreements (13). DFC can advance innovative social protection by reforming existing programs or piloting scalable initiatives (14). Moreover, integrated strategies linking climate action with socio-economic priorities enhance the effectiveness of public DFC and strengthen resilience to climate shocks by fostering healthier, more productive populations (15).

Theoretically, the link between the effects of the DFC on public health outcomes and life satisfaction can be grounded in the impacts of climate change on quality of life. Financial aid for environmental quality can influence individuals’ quality of life and satisfaction through various channels. Improvements in environmental quality influence human health both directly, by mitigating water, air, and noise pollution, as well as hazardous substances, and indirectly, by reducing the risk of natural disasters. Therefore, access to a clean environment and clean water enhances residents’ happiness and life satisfaction (11). Moreover, environmental protection expenditure and life satisfaction are directly and positively linked (13). Therefore, any climate-related policy actions, including DFC, can affect social welfare by altering countries’ environmental performance.

However, although scholars in ecological economics advise studying the influence of environmental protection spending and financial flows on wellbeing (13), empirical work on the impacts of DFC on life satisfaction and public health outcomes remains limited. Most existing empirical works focus on the effects of DFC on carbon emissions reduction (16–19), its effects on hunger alleviation and food security in Africa (12, 20), leaving a research gap for assessing the role of DFC on public health outcomes and life satisfaction of wide range of developing countries in the GS. Although the relationship between environmental policy, wellbeing, and life satisfaction has received increasing scholarly attention (21), the role of DFC in shaping public health and life satisfaction remains underexplored. Given the complex interplay between environmental protection, climate finance flows to GS countries, and human wellbeing, a key question is how DFC influences life satisfaction and public health outcomes.

Furthermore, we argue that the effectiveness of DFC in enhancing public health outcomes and life satisfaction may be affected by differences across countries in DFC receipt, driven by their level of economic development (22, 23), health sector performance, and exposure to climate risks (24). DFC may influence public health outcomes and life satisfaction differently, as climate change poses disproportionate risks to health and healthcare systems, with the most significant impacts in countries with limited health-sector funding (25). Additionally, the public health and life-satisfaction effects of DFC will vary, since countries with higher climate vulnerability face greater exposure and tend to receive more climate finance, especially for adaptation (24, 26).

Therefore, this study aims to provide a comprehensive investigation of the effects of DFC on life satisfaction and public health outcomes in developing countries in the GS. The IV-GMM model is employed, taking panel data from 69 developing countries in the GS for the period 2011 to 2021. Moreover, further investigations are conducted, controlling for differences in economic conditions, levels of environmental exposure, and the robustness of the health sector in developing countries in the GS, to examine the links between DFC, life satisfaction, and public health outcomes.

This paper has several contributions to the literature. This study aims to contribute to this emerging body of research by examining the effects of climate-related financial flow on public health and life satisfaction in the GS. First, to our knowledge, this study is among the first to investigate how DFC affects public health and life satisfaction in developing economies in the GS. Therefore, it adds a new dimension to the expanding body of research on DFC and its effects on developing economies by examining how the volume of climate-related funding is associated with public health and life satisfaction in countries in the GS.

Second, the analysis explores how this relationship changes when accounting for heterogeneity in economic status, differences in health-sector spending, and levels of exposure to climate risks. Findings that consider these factors can guide policymakers in designing inclusive strategies that leverage DFC not only to advance climate objectives but also to support public health and societal wellbeing. Third, our study employs advanced estimation techniques, such as IV-GMM, IV-2SLS, and MMQR, which provide robust findings that control for endogeneity and heterogeneity.

Fourth, this study offers several theoretical and empirical contributions to the existing body of research. On the theoretical side, it advances the triple-bottom-line framework by linking financial flows to public health and life satisfaction. Empirically, it provides concrete insights into the effects of DFC on societal life satisfaction and public health in the GS.

Finally, the findings can guide policymakers in designing strategies to improve overall wellbeing, enhance quality of life, and foster cooperation among advanced economies and developing countries.

## 2 Literature

Promoting public health and life satisfaction is vital for enhancing productivity and building resilient societies, as improved health and wellbeing foster social stability and SD amid climate challenges. Public health outcomes can be assessed through several criteria, with common indicators including mortality rate and life expectancy at birth. Specifically, the crude death rate, defined as the number of deaths per 1,000 people, is a direct indicator of a population’s health status (8). It expresses the overall likelihood of death within a population over a given period and helps evaluate national health conditions; as a result, public health outcomes are represented by the crude death rate. Similarly, life satisfaction can be represented by individuals’ self-reported life satisfaction ratings on a hypothetical ladder, where they place themselves between 0 (representing the worst life) and 10 (representing the best life possible) (27).

Moreover, DFC encompasses climate-related finance, referring to financial resources allocated to enhancing resilience to climate-related risks, reducing greenhouse gas emissions, and facilitating a transition to low-carbon, climate-resilient development pathways (28). It plays a crucial role in overcoming financial limitations to climate action, predominantly in developing countries, where significant funding gaps continue to impede the implementation of climate-focused initiatives (29). It has been given due consideration in meeting climate-related targets and objectives set under the Paris Agreement. Within this framework, SDG 17.2 sets out clear targets for DFC from developed countries to developing countries. Besides, SDG 7 promotes stronger international cooperation to expand access to clean energy and enhance energy efficiency in developing countries, encouraging investment in energy infrastructure and clean technologies. In addition, SDG 13 highlights the urgent need to mobilize financial resources to support climate change mitigation and adaptation efforts in developing countries (19).

### 2.1 Theoretical literature

The relationship between DFC and overall social welfare is grounded in a framework encompassing the central pillars of SD, including economic, environmental, and social sustainability. The United Nations (UN) and the World Bank (WB) consider it a new framework for realizing these sustainability pillars (30). DFC is a key financial instrument that supports projects that directly contribute to environmental sustainability and health outcomes by mitigating environmental risks and disasters. Moreover, the association between public health outcomes, life satisfaction, and DFC is grounded in SD theory, the carbon-resilient growth model, and the capability approach. These theories offer a more nuanced understanding of how the flow of financial resources can enhance society’s overall wellbeing.

The SD theory emphasizes the importance of balancing economic growth, social equity, and environmental sustainability to ensure long-term welfare for current and future generations (31). Therefore, this theory integrates social, economic, and environmental factors into development strategies and policy frameworks (32). According to this theory, sustainable progress can only be achieved through approaches that promote economic development while conserving environmental resources and fostering social inclusion (33). It seeks to improve resource distribution and socio-economic development to enhance quality of life, including social wellbeing and environmental quality (34). In this context, DFC, by promoting socio-economic progress and environmental quality, can positively influence life satisfaction and public health, thereby enhancing the overall social welfare in developing countries. Therefore, by channeling financial resources toward climate initiatives, DFC strengthens SD by protecting natural resources, reducing climate-related risks, and raising living standards (35). These improvements contribute to increased life satisfaction in society by enhancing the quality of life, lowering exposure to environmental hazards, and promoting more equitable social outcomes.

Furthermore, the theoretical foundations for how DFC affects public health and life satisfaction can be linked to a carbon-resilient growth model, which promotes low-emission, climate-resilient development as the most effective means of achieving lasting socio-economic and environmental benefits (36). Therefore, from these theoretical perspectives, DFC has a positive impact on public health and life satisfaction. Additionally, the effects of DFC on these outcomes can be explained using the capability approach, which provides a framework for assessing individual wellbeing beyond traditional economic measures (37). By focusing on real opportunities rather than material resources, this approach offers a strong basis for evaluating policies and actions aimed at improving human welfare. As a result, by promoting initiatives that enhance environmental quality, boost public health, and support livelihoods, DFC can significantly expand the capabilities of and communities and individuals (38), thereby improving public health and increasing life satisfaction.

### 2.2 Empirical literature and research gaps

Based on the theoretical foundations discussed above, empirical studies have examined the role of climate-related finance in different economic activities and environmental sustainability. However, most empirical works focused on the effects of DFC on carbon emissions. For example, Liu and Paan (16) analyzed the role of DFC in lowering carbon emissions and advancing SD in recipient countries, identifying a possible link between climate funding and emission reductions. The study also suggested that countries with stronger economic performance tend to gain greater benefits from DFC on emissions mitigation. Zoungrana et al. (18) investigated the impact of DFC on environmental quality across a panel of economies from 2000 to 2019, using the GMM approach. The findings suggest that DFC has a positive effect on environmental quality, consistent with the theory of financial ecology.

Yardımcı and Oskay (19) investigated the effects of green finance and financial globalization on environmental performance in Türkiye during 2001–2021. Their findings indicated that green finance contributes positively to environmental sustainability over the long term. Also, Gong et al. (17) evaluated the heterogeneous effects of DFC on ES and SW in DFC-receiving developing countries from 2002 to 2018, using the MMQR with fixed effects. The results revealed that DFC has a positive effect on environmental sustainability.

Furthermore, some studies have examined the effects of DFC on social welfare indicators. For example, Borghi et al. (25) examined the impact of DFC on health promotion and the healthcare systems’ sustainability. Likewise, applying the GMM method, Doku and Phiri (12) examined the influence of DFC on women’s hunger alleviation in 43 African countries from 2006 to 2018. The findings implied that DFC and its components significantly affect women’s hunger alleviation in SSA. Doku et al. (39) assessed the impact of Germany’s DFC on the food security of 35 African nations and found that it improves food security in these countries.

Kelly (20) investigated the effect of DFC on food security across 37 African countries from 2012 to 2021, using system GMM. The findings showed that DFC significantly reduces food insecurity in Africa. Additionally, using the two-step system GMM model and fixed-effects Driscoll and Kraay regressions, Kelly (40) examined the impact of DFC on subjective wellbeing in 37 African countries between 2012 and 2021. The results indicated that DFC has a positive effect on life satisfaction. Moreover, a recent study by Zhao et al. (41) found that DFC significantly mitigates welfare losses from energy poverty, suggesting that increasing climate finance is a key strategy to mitigate the adverse effects of energy poverty.

The empirical work has been extended to other economic sectors. Fang et al. (23) investigated the effects of DFC on primary, energy, and water sectors using worldwide panel data. The findings reveal that DFC can increase power output and boost agricultural value added in recipient nations. Additionally, using the GMM technique on panel data for 86 recipient countries over 2002–2021, Kabutey et al. (42) assessed the effects of DFC on the business environment and found a weak positive impact. Li and Wu (28) investigated the impact of green finance on SD indices across distinct groups of developing countries. They found a positive link between green finance and sustainability. Moreover, Donath and Oprea (43) provided evidence that financial inclusion is a key element in the fight against poverty and the pursuit of inclusive development (as a prerequisite of wellbeing and happiness). Additionally, Tran et al. (44) investigated the effectiveness of climate-related financial policies in advancing the SDGs across a panel of countries. They argued that it has the potential to foster education quality, food security, wellbeing, clean energy, innovation, and peace.

Moreover, the effects of DFC on public health and life satisfaction can be heterogeneous, depending on income levels, the development of the health sector, and exposure to climate risks. For example, Lee et al. (22) showed that financial support for climate initiatives enhances environmental sustainability in economically better-income economies. Also, Weiler et al. (45) demonstrated that nations with better income tend to attract more climate-related funding than lower-income nations, suggesting that the role of DFC varies across receiving countries. The heterogeneity analysis of Fang et al. (23) indicated that differences in income significantly affect the effectiveness of DFC across sectors. Also, Liu et al. (24) found that countries more vulnerable to climate change tend to receive higher levels of both mitigation and adaptation funding. Consistent with Liu et al. (24) and Prasad and Singhania (26) found strong alignment with climate justice principles: countries with higher climate vulnerability receive greater levels of DFC.

## 3 Methodology and brief on data

### 3.1 Model construction and hypotheses development

By strengthening resilience to climate-related risks and facilitating a transition toward low-carbon, climate-resilient development pathways, DFC can improve public health and life satisfaction. In line with SD Theory, advancing balanced economic growth, social equity, and environmental protection supports long-term wellbeing for both present and future generations, thereby enhancing overall social welfare. Moreover, it aims to enhance socioeconomic development and resource redistribution to improve the quality of life, including social and environmental wellbeing (34). In this context, DFC can enhance public health and life satisfaction in developing countries by promoting both socioeconomic development and environmental quality. It contributes to improved quality of life, reduced exposure to environmental risks, and more equitable social outcomes.

By supporting initiatives that strengthen environmental conditions and livelihoods, DFC can significantly expand individuals’ and communities’ capabilities (38), thereby positively influencing life satisfaction and public health. Supporting these arguments, DFC contributes to hunger alleviation (12), enhances food security (20, 39), boosts subjective wellbeing and reduces welfare losses (40, 41), promotes SD (44), and strengthens the sustainability of healthcare systems (25), all of which positively impact overall social welfare. Thus, the following hypothesis is formulated. The study framework and details of hypothesis development are depicted in Supplementary Figure S1.

*Hypothesis 1A (H1A)*: DFC positively affects public health outcomes in developing countries in the GS.

*Hypothesis 1B (H1B)*: DFC positively affects life satisfaction in developing countries in the GS.

Therefore, the crude death rate, used to measure public health, defined in Equation 1, will be negatively influenced by DFC ( $\alpha_{1}=\partial \text{Crudedeat}h_{\mathrm{it}}/\partial \mathrm{DF}C_{\mathrm{it}}<0$ ). Likewise, life satisfaction in Equation 2 will be positively affected by DFC ( $\beta_{1}=\partial \text{Lsatisfactio}n_{\mathrm{it}}/\partial \mathrm{DF}C_{\mathrm{it}}>0$ ).

$$\text{Crudedeat}h_{\mathrm{it}}=\alpha_{0}+\alpha_{1}\mathrm{DF}C_{\mathrm{it}}+\alpha_{n}\text{Control}s_{\mathrm{it}}+\lambda_{\mathrm{it}}$$ (1)

$$\text{Lsatisfactio}n_{\mathrm{it}}=\beta_{0}+\beta_{1}\mathrm{DF}C_{\mathrm{it}}+\beta_{n}\text{Control}s_{\mathrm{it}}+\eta_{\mathrm{it}}$$ (2)

Where *Crudedeath* represents crude death rate, *Lsatisfaction* is life satisfaction, *DFC* is development finance for climate, Controls represent control variables such as *industry* (value added, % of GDP), *urban* (population residing in urban areas, % of total population), *growth* (real GDP per capita growth rate), *trade* (merchandize, % GDP), *FDI* (the net inflow of FDI, % of GDP), *health* (health expenditure, % of GDP), *digital* (aggregate indicator of digital infrastructure), and *quality of institutions index (QI)* (aggregate indicator of quality of institutions).

Additionally, the effects of DFC on public health and life satisfaction are shaped by differences in economic development, health-sector capacity, and exposure to environmental risks of developing countries, as climate change disproportionately impacts developing countries with limited health sector financing (25) and more climate-vulnerable countries (24, 26). Besides, income influences the effectiveness of DFC (22). The following hypotheses are tested.

*Hypothesis 2A (H2A)*: The effects of DFC on public health outcomes are heterogeneous, subject to differences in the level of income, health sector development, and level of exposure to climate risks.

*Hypothesis 2B (H2B)*: The effects of DFC on life satisfaction are heterogeneous, subject to differences in the level of income, health sector development, and level of exposure to climate risks.

### 3.2 Data and variables

This research focuses on 69 Developing countries in the GS for the period 2011 to 2021, given the availability of data on the major variables.^1^

*Dependent variables*: There are two dependent variables: crude death rate to represent public health, and life satisfaction. The crude death rate, defined as the number of deaths per 1,000 people, is a direct indicator of a population’s health status (8, 46) and represents public health outcomes. It expresses the overall risk of death in a population over a given period and helps assess national health conditions. Because it captures deaths from all causes, the crude death rate reflects a range of influences on population health, including disease outbreaks, healthcare quality, lifestyle factors, and environmental conditions. Consequently, it is also valuable for comparing public health levels across countries (9). Because the crude death rate offers a transparent, easily interpretable measure and responds more quickly to public health emergencies or crises, this study adopts the crude death rate as its primary indicator. Alternatively, for a robustness test, life expectancy is used to represent public health. It serves as a key measure of a nation’s public health status, representing the average number of years a newborn is expected to live assuming current mortality patterns persist (47). This indicator reflects the combined effects of numerous health determinants, such as socioeconomic conditions, healthcare accessibility, and environmental quality. Higher life expectancy generally signals a strong public health system capable of preventing illness and promoting wellbeing, while a decline may indicate emerging health risks or strain on medical services.

Moreover, life satisfaction is measured using individuals’ self-reported life satisfaction on a hypothetical ladder, where they place themselves between 0 (representing the worst life they could imagine) and 10 (representing the best life possible). It is the most frequently used and best-validated concept for measuring wellbeing (48, 49). The data are drawn from the OurWorld in Data database, which provides a consistent cross-country measure of subjective wellbeing (27). This study used the life satisfaction indicator, represented by the Cantril ladder self-reported life satisfaction, following studies such as Kelly (40) and Yan et al. (11).

*Target variable*: DFC has emerged as a crucial tool in supporting worldwide initiatives to both reduce and adapt to the impacts of climate change. It includes financial flows designed to cut greenhouse gas emissions, strengthen resilience to climate-related risks, and support the shift toward low-carbon, climate-resilient development pathways (28). This study uses data on DFC from the OECD DFC project database, which covers more than 188,000 projects implemented between 2000 and 2021 across sectors such as energy, agriculture, and health. This study uses DFC data from 2011 to 2021 to explore its potential impact on life satisfaction and public health, since life satisfaction indicator data have been available since 2011.

*Control variables*: When examining how DFC affects life satisfaction and public health, it is essential to include appropriate controls to ensure the findings are accurate and reliable. Based on existing empirical and theoretical foundations, urbanization (49), industrialization, trade, FDI, health spending, digital infrastructure (50), and IQ (51). Details about each variable are presented in Table 1.

**Table 1:** Details about major variables.

Variables	Abbreviations	Definition	Data sources
Public health outcomes	Crudedeath	Proxied by the crude death rate (the number of deaths per 1,000 people).	The WB database: https://data.worldbank.org/indicator/SP.DYN.CDRT.IN
Life satisfaction (Lsatisfaction)	Lsatisfaction	Measured using the Cantril Ladder self-reported life satisfaction index. Its scale is from 0 (lowest) to 10 (highest).	Our World in Data database: https://ourworldindata.org/grapher/happiness-cantril-ladder
Development finance for climate and the environment	DFC	Climate mitigation and adaptation financial aid from advanced economies to developing countries.	OECD climate finance project database: https://www.oecd.org/en/topics/sub-issues/development-finance-for-climate-and-the-environment.html
Real GDP per capita growth	growth	Real GDP per capita growth rate.	WDI database: https://databank.worldbank.org/source/world-development-indicators
Net inflow of FDI	FDI	The net inflow of FDI in developing countries (% of GDP).	WDI datasets: https://databank.worldbank.org/source/world-development-indicators
Industrialization	industry	Industry (including construction), value added (% of GDP)	https://data.worldbank.org/indicator/NV.IND.TOTL.ZS
Health spending	health	Health expenditure (% of GDP)	https://data.worldbank.org/indicator/SH.XPD.CHEX.GD.ZS
Urbanization	urban	Population residing in urban areas (% of total population)	https://databank.worldbank.org/source/world-development-indicators
Institutional quality	IQ	Aggregate IQ index derived using control of corruption, regulatory quality, rule of law, government effectiveness, absence of violence and terrorism and voice and accountability.	available from WB WGI^1^: https://www.worldbank.org/en/publication/worldwide-governance-indicators
Digitalization	digital	Aggregate indicator of digital infrastructure derived using mobile cellular subscriptions, broadband subscriptions, and internet usage, applying PCA strategy.	WDI database: https://databank.worldbank.org/source/world-development-indicators

^1^Aggregate indicators for quality of institutions and digital infrastructure are derived employing principal component analysis. The results will be provided upon request.

### 3.3 Method of analysis

There may be reverse causality between the dependent and independent variables. For example, changes in public health and life satisfaction of DFC-receiving countries will affect the flow patterns of DFC to developing countries. Conversely, financial flows from developed to developing countries affect public health outcomes and life satisfaction. This bidirectional relationship may create simultaneity, leading to biased coefficients when using OLS and other conventional panel regression methods (52). Therefore, OLS and other conventional estimators for this model do not address endogeneity. Failure to account for endogeneity can lead to biased regression results and erroneous conclusions. However, it is also challenging to find a pure, exogenous, and strong external instrument of the DFC. Specifically, this instrument should have a robust relationship with DFC but no relationship with the error term, and it should be related only to public health outcomes and life satisfaction via DFC.

Therefore, to address endogeneity, the IV-GMM method is employed, as it is a robust approach for controlling for potential endogeneity (53). This method addresses estimation issues related to endogeneity, omitted variable bias, and measurement error, thereby enhancing the reliability and consistency of the results (52, 54). Hence, it is the most efficient and consistent choice for addressing endogeneity problems such as reverse causality, measurement errors, and omitted-variable bias (55, 56).

Besides, this estimation technique is robust to autocorrelation and yields more reliable estimates when unaccounted heteroskedasticity is present (57). It also accounts for heteroscedasticity and autocorrelation. It is particularly well-suited for datasets with more cross-sectional units than time periods [N (58)> T (11)]. Furthermore, even when heteroscedasticity is uncertain, IV-GMM can effectively address omitted-variable bias, yield consistent estimates, and deliver satisfactory results. Accordingly, the IV-GMM is applied to estimate the following log-linear equations, thereby controlling for potential endogeneity concerns.

$$\begin{array}{l} \begin{array}{l} \text{Crudedeat}h_{\mathrm{it}}=\alpha_{0}+\alpha_{1}\mathrm{DF}C_{\mathrm{it}}+\alpha_{2}\text{industr}y_{\mathrm{it}}+\alpha_{3}\text{urba}n_{\mathrm{it}}\\ +\alpha_{4}\text{growth}+\alpha_{5}\text{trad}e_{\mathrm{it}}+\alpha_{6}\mathrm{FD}I_{\mathrm{it}}+\alpha_{7}\text{healt}h_{\mathrm{it}}+\alpha_{8}\text{digita}l_{\mathrm{it}} \end{array}\\ +\alpha_{8}QI_{\mathrm{it}}+\lambda_{\mathrm{it}}+\gamma_{t} \end{array}$$ (3)

$$\begin{array}{l} \begin{array}{l} \text{Lsatisfactio}n_{\mathrm{it}}=\beta_{0}+\beta_{1}\mathrm{DF}C_{\mathrm{it}}+\beta_{2}\text{industr}y_{\mathrm{it}}+\beta_{3}\text{urba}n_{\mathrm{it}}\\ +\beta_{4}\text{growt}h_{\mathrm{it}}+\beta_{5}\text{trad}e_{\mathrm{it}}+\;\beta_{6}\mathrm{FD}I_{\mathrm{it}}+\beta_{7}\text{healt}h_{\mathrm{it}}+\beta_{8}\text{digita}l_{\mathrm{it}} \end{array}\\ +\beta_{9}QI_{\mathrm{it}}+\eta_{\mathrm{it}}+\gamma_{t} \end{array}$$ (4)

Where, η_it_ and λ_it_ represent stochastic terms and γ_t_ is time fixed effects. The crude death rate and DFC have been transformed using natural logarithms, whereas the remaining variables have not, as they are either ordinal or expressed as percentages.

Finally, robustness checks were performed using the method of moments quantile regression (MMQR), two-step system generalized method of moments (GMM), feasible generalized least squares (FGLS), maximum likelihood estimation (MLE), and generalized structural equation modeling (GSEM). Additional robustness analysis is conducted using life expectance, which is an alternative proxy for public health outcomes.

## 4 Findings and discussions

### 4.1 Preliminary analysis

Summary statistics of the major variables are reported in Table 2. Similarly, the correlation coefficients for the variables included in the analysis are reported in Table 3, indicating no high correlation among the explanatory variables. Moreover, a multicollinearity test is conducted to assess multicollinearity concerns, and, consistent with the correlation analysis, the results in Supplementary Table S1 indicate that there are no multicollinearity issues.

**Table 2:** Summary of variables.

Variable	Obs	Mean	Std. dev.	Min	Max	JB
Crudedeath_it_	759	7.116	1.986	3.033	18.387	2.431
Lsatisfaction_it_	751	5.041	0.878	2.839	7.257	12.450
DFC_it_	747	5.28E+05	9.76E+05	1.750	1.10E+07	1113.000
industry_it_	759	29.320	10.014	2.759	79.961	361.000
urban_it_	759	54.319	20.604	16.208	95.603	68.150
growth_it_	759	3.703	6.045	−50.339	86.827	4.5e+06
trade_it_	759	56.158	28.658	15.208	182.563	11.030
FDI_it_	751	3.585	5.271	−37.173	43.912	3.0e+06
health_it_	759	5.475	2.082	1.752	11.009	25.480
digital_it_	738	0.00	8.000	1.474	−2.926	4.342
QI_it_	759	0.00	9.000	2.129	−5.199	6.371

**Table 3:** Correlation coefficients.

Variables	a	b	c	d	e	f	g	h	i	j
Panel I: Correlation coefficients for variables included in the link between DFC and public health										
Crudedeath_it_	1									
DFC_it_	−0.041	1								
industry_it_	−0.193	−0.157	1							
urban_it_	−0.244	−0.119	0.217	1						
growth_it_	0.018	−0.011	0.125	−0.229	1					
trade_it_	−0.159	−0.278	0.257	0.117	0.063	1				
FDI_it_	0.000	−0.035	−0.054	−0.066	0.127	0.149	1			
health_it_	−0.139	−0.049	−0.270	0.309	−0.219	0.125	0.062	1		
digital_it_	−0.216	0.049	0.069	0.620	−0.255	0.133	−0.038	0.467	1	
IQ_it_	−0.113	0.043	−0.157	0.320	−0.059	0.158	0.011	0.359	0.590	1
Panel II: Correlation coefficients for variables included in the link between DFC and life satisfaction										
Lsatisfaction_it_	1									
DFC_it_	0.048	1								
industry_it_	0.084	−0.160	1							
urban_it_	0.540	−0.118	0.211	1						
growth_it_	−0.099	−0.007	0.135	−0.227	1					
trade_it_	0.069	−0.281	0.249	0.117	0.063	1				
FDI_it_	0.042	−0.032	−0.042	−0.061	0.119	0.155	1			
health_it_	0.374	−0.048	−0.255	0.322	−0.231	0.133	0.051	1		
digital_it_	0.620	0.051	0.082	0.631	−0.266	0.136	−0.047	0.461	1	
IQ_it_	0.354	0.047	−0.148	0.327	−0.068	0.161	0.001	0.352	0.587	1

### 4.2 Baseline findings

Baseline results on the effects of DFC on public health and life satisfaction are reported in Table 4. Column (I) reports findings of the effects of DFC on public health outcomes, and Column (II) presents findings of the effects of DFC on life satisfaction. Model diagnostic tests, such as Kleibergen-Paap rk LM statistic (KP LM-statistic), Kleibergen-Paap rk Wald F statistic (KP F-statistic), and Hansen J statistic (HJ-statistic), indicate that the IV-GMM model exhibits no under identification, weak identification, or overidentification.

**Table 4:** The role of DFC on crude death rate and life satisfaction.

Variables	(I) (Crude death rate)	(II) (Life satisfaction)
DFC_it_	−0.025*** (0.006)	0.047*** (0.015)
industry_it_	−0.005*** (0.001)	0.001 (0.005)
urban_it_	−0.003*** (0.001)	0.007*** (0.002)
growth_it_	−0.005* (0.003)	0.020** (0.009)
trade_it_	−0.001*** (0.000)	−0.002* (0.001)
FDI_it_	0.001 (0.002)	0.017*** (0.005)
health_it_	−0.010 (0.006)	0.081*** (0.017)
digital_it_	0.007 (0.014)	0.365*** (0.034)
IQ_it_	−0.003 (0.007)	−0.041* (0.021)
_cons	2.844*** (0.130)	3.140*** (0.314)
Time FE	Yes	Yes
Obs.	602	596
R^2^	0.465	0.514
KP LM-statistic	69.580	66.695
KP F-statistic	72.606	71.450
HJ-statistic	0.290	0.351

* < 0.1, ** < 0.05, *** < 0.01, standard errors in brackets.

The findings in Table 4 show that urbanization has a significant adverse effect on crude death rate and a positive effect on self-reported life satisfaction. Therefore, this result implies that promoting urbanization in developing countries in the GS positively affects public health outcomes and life satisfaction in the developing countries of the GS. The findings are consistent with prior research indicating that urban areas tend to enhance subjective wellbeing through convenient amenities, such as parks, shopping centers, and transportation networks, and high-quality services, including public safety, education, and healthcare (59). Consequently, urban areas offer residents a more supportive living environment, and the urban built environment creates spaces for social interaction and contributes to better physical and mental health (59).

Moreover, the results imply that digitalization has a positive effect on life satisfaction in developing countries in the GS. The findings can be loosely explained as digital infrastructure improves life satisfaction in developing countries by creating economic opportunities, enhancing access to health and education, and strengthening social connections, positively affecting life satisfaction. It provides tools to increase income through new agricultural technologies and entrepreneurship, while also enabling remote learning and telemedicine and fostering social capital through improved communication. Moreover, the findings imply that health spending in developing countries in the GS positively influences life satisfaction.

Likewise, economic size (GDP growth) has a negative effect on crude death rate and a positive effect on life satisfaction. Growth improves individuals’ incomes and living standards, which tends to improve public health outcomes and life satisfaction. Similarly, FDI inflows positively affect life satisfaction in developing countries in the GS. However, IQ has an adverse effect on life satisfaction, though the effect is weakly significant.

In addition, the coefficient on industrialization is negative in Column (I), indicating a negative association between industrialization and crude death rate; hence, it has a positive effect on public health outcomes in developing countries in the GS. The positive relationship between industrialization and public health is supported by structural change theory, which posits that as economies transition from agriculture to industry, job creation, income levels, and overall societal welfare improve, particularly in developing countries (60). Likewise, trade openness negatively affects crude death rate, positively contributing to public health outcomes. It has a negative effect on life satisfaction at 10% of level of significance.

Regarding the target variable, the results indicate a significant adverse effect on crude death rate and a significant positive effect on life satisfaction, confirming that increased DFC contributes positively to public health outcomes and life satisfaction in developing countries in the GS. Specifically, a 1% increase in DFC inflows to developing countries in the GS is associated with a 0.025% decline in crude death rate (Table 4). This demonstrates that DFC flows into socio-economic and environmental initiatives effectively enhance public health in recipient nations in the GS. These findings are arguable as targeted DFC strengthens a country’s healthcare capacity and service quality by expanding medical infrastructure and contributes to cleaner air, safer drinking water, lower rates of infectious diseases, and overall improvements in population health (61). These results align with the empirical evidence presented by Khan et al. (62) and support the hypothesis proposed by Borghi et al. (25). Moreover, it can enhance public health outcomes and life satisfaction by contributing to greater societal wellbeing through support for SD and reduced climate-related vulnerabilities (13).

Furthermore, DFC has a positive role in enhancing life satisfaction in developing countries in the GS. The results imply that a 1% increase in DFC inflows to developing countries in the GS is associated with a 0.047-unit increase in the life satisfaction score (Table 4). The findings are consistent with those of Kelly et al. (40), who reported similar results for African countries. Therefore, an increase in DFC can reinforce funding for projects aimed at mitigating the adverse effects of climate change, such as floods, droughts, and extreme weather events, which particularly affect developing countries in the GS. Financial support for investments in renewable energy, sustainable farming practices, and climate-resilient infrastructure in developing countries can help improve food security, promote greater economic stability, and lead to better health outcomes (20, 63). In turn, these advances contribute to alleviating poverty and reducing vulnerability, fostering social wellbeing, which collectively boosts overall life satisfaction (40). By contributing to improved environmental quality, which is shown to shape quality of life (64), DFC positively impacts life satisfaction. To sum up, DFC enhances resilience and environmental quality in developing countries in the GS by reducing vulnerability to climate risks, enhancing economic stability, and ultimately increasing overall life satisfaction.

### 4.3 Robustness analysis

First, to control for heterogeneity across countries in the distribution of crude death rate and life satisfaction, the analysis is repeated using the MMQR. For example, the average life satisfaction and crude death rate range from 2.8 to 7.3 and from 3.033 to 18.387, respectively, indicating substantial variation across the DC sample (Table 2). Similarly, there is heterogeneity among countries as indicated by the normality test (Table 2). These conditions call for an alternative model that captures the heterogeneity of the dependent variables (65). Thus, the MMQR approach is used to control for distributional heterogeneity. Given the data’s distributional characteristics, the analysis is re-estimated using the MMQR to account for distributional heterogeneity.

The results show that DFC has a robust adverse effect on crude death rate across all quantiles, implying that DFC positively contributes to public health outcomes across all quantiles in the sample of countries. Therefore, the negative relationship between DFC and the death rate in developing countries implies that increased climate-related funding effectively reduces mortality in the GS, thereby improving public health outcomes. The findings further imply that this impact is more substantial in nations with higher death rates, suggesting DFC enhances resilience, healthcare access, and adaptive capacity where vulnerabilities are most severe. The rising coefficient of DFC from lower to higher death-rate quantiles indicates that DFC has a greater impact at higher mortality levels (Table 5). This suggests that the DFC flow is more effective at reducing deaths among the most vulnerable populations, highlighting its targeted benefits in high-risk contexts.

**Table 5:** The effects of DFC on crude death rate and life satisfaction.

Variables	Q.1	Q.25	Q.5	Q.75	Q.9
Panel I: The link between DFC and crude death rate					
logDFC_it_	−0.013** (0.005)	−0.016*** (0.005)	−0.021*** (0.004)	−0.027*** (0.005)	−0.031*** (0.005)
Controls	Yes	Yes	Yes	Yes	Yes
Obs.	731	731	731	731	731
Panel II: The effects of DFC on life satisfaction					
logDFC_it_	0.079*** (0.019)	0.063*** (0.015)	0.043*** (0.011)	0.027*** (0.010)	0.012 (0.012)
Controls	Yes	Yes	Yes	Yes	Yes
Obs.	723	723	723	723	723

** < 0.05, *** < 0.01.

Moreover, the findings implied that DFC has a heterogeneous effect on life satisfaction, with a more pronounced effect in countries with lower life satisfaction indices. Therefore, the findings imply that DFC has more potent effects in countries with lower life-satisfaction indices because these nations often face greater vulnerabilities, poorer health, weaker infrastructure, and limited resources. Targeted climate investments directly address these deficits, producing larger improvements in wellbeing than in countries with higher baseline life satisfaction.

Second, alternatively, life expectancy at birth is used to represent public health, based on data from the Our World in Data database.^2^ The findings in Table 6 imply that DFC has a positive and significant effect on life expectancy, confirming the baseline findings reported in Column (I) in Table 4.

**Table 6:** The effects of DFC on life expectancy.

Variables	(I)
DFC_it_	0.006*** (0.001)
Control variables	Yes
Time FE	Yes
Obs.	602
R2	0.600
KP LM-statistic	88.841
KP F-statistic	247.406
HJ-statistic	0.112

*** < 0.01.

Third, the FGLS approach is used to further address concerns about heterogeneity and serial correlation. In the presence of cross-sectional dependence, FGLS yields more efficient estimates than methods that assume independence across panels (66). Besides, the MLE is used to address potential sample bias. Consistent with the baseline results in Table 4, the FGLS and MLE findings indicate that DFC has a robust negative effect on the crude death rate and a significant positive effect on life satisfaction in developing countries in the GS (see Supplementary Tables S2, S3).

Fourth, the two-step system GMM approach is used as it employs additional instruments for the lagged dependent variable and other endogenous regressors, thereby improving estimation efficiency and addressing endogeneity (67). The findings indicate that DFC has a negative effect on crude death rate and a positive effect on life satisfaction (Supplementary Table S4).

Finally, following the work of Danish and Khan (68), this study applies a generalized structural equation model (GSEM) as a convenient strategy to estimate direct and indirect effects, incorporating mediation analysis. Therefore, using GSME, further analysis is conducted to evaluate the mediating role of crude death rate in the association between life satisfaction and DFC. The indirect effect is significant, confirming the mediating role of the crude death rate (Supplementary Table S5). The findings indicate that DFC’s effect is partially mediated by crude death rate, as its effect on life satisfaction decreases but remains statistically significant after crude death rate is included. The mediation further illustrated by Supplementary Figure S2.

### 4.4 The role of the health sector

The analysis further examines how differences in domestic financial capacity within the health sector influence the effects of DFC on public health and life satisfaction. Hence, using the average value of health expenditure, developing countries in the GS are classified as having either lower or higher health expenditure, based on the sample countries’ mean health expenditure (Table 7).

**Table 7:** The role of differences in health spending.

Variables	(I) (Crude death rate)	(II) (Life satisfaction)
Panel I: Countries with the health spending greater than the mean		
logDFC_it_	−0.0142* (0.008)	0.048** (0.022)
Control variables	Yes	Yes
Time FE	Yes	Yes
Obs.	245	244
R2	0.447	0.479
KP LM-statistic	17.354	17.377
KP F-statistic	195.546	190.145
HJ-statistic	0.166	0.091
Panel II: Countries with health spending less than the mean value		
logDFC_it_	−0.030*** (0.007)	0.0643*** (0.212)
Control variables	Yes	Yes
Time FE	Yes	Yes
Obs.	311	306
R2	0.327	0.421
KP LM-statistic	72.372	70.022
KP F-statistic	89.80	87.497
HJ-statistic	0.315	0.855

* < 0.1, ** < 0.05, *** < 0.01.

The findings in Table 7 indicate that the effects of DFC on crude death rate are robustly negative across developing countries in the GS. However, the coefficient of DFC is higher for developing countries with lower health-sector expenditure. Additionally, DFC has a positive effect on life satisfaction in countries with both higher and lower health-sector expenditure. However, the magnitude of the DFC coefficient is higher in countries with lower health-sector expenditure. Therefore, the findings show that developing countries with lower health expenditure benefit more from DFC in enhancing life satisfaction and improving public health outcomes in the GS. This is because DFC tends to have more potent effects in nations with limited medical infrastructure and public health capacity. Consequently, additional climate funding from advanced economies helps bridge critical gaps by improving access to healthcare, disease prevention, and resilience to climate shocks. Therefore, DFC flow helps address gaps in infrastructure and service capacity, leading to notable improvements in population health (69).

Conversely, countries with higher health spending already possess more robust systems, resulting in a smaller marginal impact of DFC on public health and life satisfaction. Similarly, DFC significantly increases life satisfaction in developing countries with lower health expenditure by directly improving essential health services, resilience, and living conditions. In contrast, nations that already meet basic needs due to higher health expenditure experience smaller gains in life satisfaction from additional climate funding. By comparison, in countries where public health systems are already well developed, additional climate-related funding may have limited room to generate substantial further gains (58).

### 4.5 The role of economic conditions

To assess how differences in economic development levels affect the effectiveness of DFC on public health outcomes and life satisfaction, the sample countries are classified as low-income or middle-income based on the World Bank’s country classifications. The findings show that in middle-income countries, DFC exerts a clear and significant positive influence on public health outcomes and life satisfaction. In contrast, this beneficial effect is far less evident in low-income countries in the GS. A plausible explanation is that countries with better income levels benefit more from DFC to enhance public health and life satisfaction. These results can be loosely explained by the fact that the impacts of DFC on public health and life satisfaction are greater in middle-income economies because these countries possess stronger institutional capacity, governance, and infrastructure for effective use of funds. They can more efficiently integrate climate investments into health systems and development plans, resulting in stronger outcomes. In contrast, low-income economies often face structural constraints that limit the effectiveness of implementation (Table 8).

**Table 8:** The role of economic size.

Variables	(I)	(II)
Panel I: Low-income		
logDFC_it_	−0.089 (0.056)	0.109 (0.098)
Control variables	Yes	Yes
Time FE	Yes	Yes
Obs.	0.492	70
R2	0.492	0.770
KP LM-statistic	19.082	9.93
KP F-statistic	21.770	16.001
HJ-statistic	0.810	0.880
Panel II: Middle income		
logDFC_it_	−0.018*** (0.007)	0.042*** (0.015)
Control variables	Yes	Yes
Time FE	Yes	Yes
Obs.	532	526
R2	0.242	0.472
KP LM-statistic	64.409	61.659
KP F-statistic	155.238	161.538
HJ-statistic	0.332	0.467

*** < 0.01.

### 4.6 Level of exposure to environmental-related risks

To test the argument that the effects of DFC on public health and life satisfaction may be heterogeneous, depending on the level of exposure to environmental-related risks, the sample countries are categorized as having low or high exposure to such risks. For this purpose, we used population exposure to fine particulate matter (PM2.5), which penetrates deep into the lungs, increases the risk of heart disease, stroke, and premature death. The results are reported below (Table 9).

**Table 9:** The role of differences in environmental-related risks.

Variables	I	II
Panel I: high exposure		
logDFC_it_	−0.027*** (0.007)	0.064** (0.025)
Control variables	Yes	Yes
Time FE	Yes	Yes
Obs.	318	317
R2	0.244	0.337
KP LM-statistic	113.436	114.716
KP F-statistic	422.483	456.832
HJ-statistic	0.503	0.767
Panel II: Low-exposure		
logDFC_it_	−0.024*** (0.009)	0.001 (0.017)
Control variables	Yes	Yes
Time FE	Yes	Yes
Obs.	266	261
R2	0.259	0.737
KP LM-statistic	13.787	13.923
KP F-statistic	103.582	111.574
HJ-statistic	0.173	0.284

** < 0.05, *** < 0.01.

The findings show that DFC has a significant adverse effect on crude death rates in developing countries in the GS, regardless of exposure to environmental risks. Nonetheless, the magnitude of the DFC coefficient is greater in countries with higher exposure to environmental risks, implying that it has a greater impact on crude death rate in such countries. Moreover, it has a positive effect on life satisfaction in countries with greater exposure to environmental risks. Therefore, the results imply that DFC has a greater effect on reducing mortality and promoting life satisfaction in developing countries with greater exposure to climate-related health risks. DFC exerts a greater positive effect on public health in countries with higher PM2.5 concentrations because these nations face more severe air pollution–related health challenges, including respiratory and cardiovascular diseases. DFC directed toward clean energy, emission reduction, and environmental management directly lowers pollution levels and health risks. As a result, the marginal benefits of climate investments are larger where pollution is most acute, leading to substantial improvements in air quality, disease prevention, and overall population health.

## 5 Conclusions and policy implications

This study examines the impact of DFC on public health outcomes and life satisfaction in developing countries across the GS, drawing on panel data from 69 countries for 2011–2021. To address potential endogeneity concerns, the analysis employs an IV-GMM estimator. It provides comprehensive empirical evidence on the role of DFC in improving public health outcomes and life satisfaction in developing countries of the GS. The findings are summarized as follows.

First, the findings demonstrate that DFC positively influences public health outcomes in developing countries in the GS. Therefore, DFC flow generates benefits that extend well beyond environmental protection, contributing meaningfully to human wellbeing and social welfare by promoting public health outcomes. Hence, the positive effects of DFC on public health outcomes underscore its critical role in strengthening health systems, reducing vulnerability to climate-sensitive diseases, and improving access to essential services. Therefore, DFC, by supporting climate-resilient infrastructure, clean energy, water and sanitation, and disaster preparedness, appears to yield tangible health gains, promoting public health outcomes in developing countries in the GS.

Second, the results show that DFC positively affects life satisfaction in developing countries in the GS, implying that DFC enhances subjective wellbeing by reducing environmental quality, improving living conditions, and fostering a sense of security and resilience among populations. Therefore, the finding underscores the importance of prioritizing climate-vulnerable countries in global financing mechanisms to maximize public health outcomes and life satisfaction.

Third, the study’s findings reveal substantial heterogeneity in the effects of DFC on public health outcomes and life satisfaction. More specifically, developing countries with lower levels of health expenditure benefit disproportionately, indicating that DFC can serve as a complementary resource to fill domestic health spending gaps. Thus, DFC flows may help close critical infrastructure and service gaps, deliver health and wellbeing improvements, promote public health outcomes, and life satisfaction in developing countries with lower levels of health expenditure. Moreover, the findings further show that developing countries with better income levels in the GS are better able to translate DFC into improved public health outcomes and life satisfaction. This suggests that developing countries with better income levels will have stronger institutional capacity, stronger administrative systems, complementary investments, and economic stability, which can play an important role in determining the effectiveness of DFC.

Finally, the heterogeneity analysis further provides evidence that developing countries with greater exposure to environmental and climate-related risks experience larger positive effects of DFC on promoting public health outcomes and life satisfaction. Therefore, developing countries with more exposure to climate-related risks appear to enhance adaptive capacity, reduce health risks from extreme weather events, and mitigate the adverse impacts of environmental degradation on daily life, improving public health outcomes and life satisfaction.

Therefore, this research contributes to the growing literature by explicitly linking DFC to both public health outcomes and subjective wellbeing, providing comprehensive evidence that DFC should be viewed not only as an environmental or economic tool but also as a crucial investment in public health outcomes and life satisfaction. Thus, it has important policy implications for governments, policymakers, and stakeholders.

First, the findings confirm that increased DFC inflows are associated with improved public health indicators and higher life satisfaction, underscoring the importance of integrating DFC into broader development and health strategies. It is recommended that public health outcomes and life satisfaction be integrated into DFC flow criteria to ensure that funded projects directly contribute to improved living conditions and subjective wellbeing. Therefore, policymakers and international donors should align DFC strategies with public health and social development goals. Doing so can enhance the effectiveness of climate finance and support more inclusive and sustainable development pathways in the GS.

Second, the findings indicated that DFC has more effects on public health outcomes and life satisfaction in developing countries in the GS with more exposure to environmental-related risk. Therefore, greater focus should be placed on these countries, which often lack resources and adaptive capacity, to ensure that climate finance addresses critical gaps and yields noticeable improvements in resilience, public health, and wellbeing. Therefore, it calls for ensuring that the flow of financial resources is targeted toward vulnerable populations and high-risk countries.

Third, the findings evidenced that developing countries with financial gaps in the health sector benefit more from DFC to enhance public health outcomes and life satisfaction. Therefore, governments should treat environmental spending as a key policy tool for enhancing domestic wellbeing, underscoring the crucial link between environmental quality and overall welfare and life satisfaction.

Finally, the findings of this study provide comprehensive evidence on the effects of DFC on public health outcomes and life satisfaction. However, the study has some limitations that future research should consider. The effects of DFC on public health outcomes and life satisfaction often manifest over longer periods, whereas the 2011–2021 timeframe used in this study may be insufficient to capture their long-term effects fully. Additionally, this study considered 69 developing countries in the GS due to data availability, which may limit the general applicability of the results to other developing or least-developed countries. Thus, future research, whenever data are available, should consider a longer time horizon and a wider range of developing countries in the analysis of the association among DFC, public health, and life satisfaction. Moreover, the aggregate DFC is used in this study. However, future work should extend the analysis by disaggregating DFC by sector, as sectoral allocations of DFC may have varying effects on public health and life satisfaction.

## Data Availability

The raw data supporting the conclusions of this article will be made available by the authors, without undue reservation.

## Footnotes

1 List of countries: Angola, Algeria, Argentina, Bangladesh, Benin, Bolivia, Botswana, Bosnia and Herzegovina, Brazil, Cameroon, Cambodia, China, Chile, Colombia, Congo Dem., Congo Rep., Côte d’Ivoire, Costa Rica, Dominican Rep., Ecuador, El Salvador, Egypt, Ethiopia, Ghana, Gabon, Guatemala, Haiti, Honduras, India, Indonesia, Iraq, Iran, Jamaica, Jordan, Kenya, Laos, Libya, Lebanon, Madagascar, Mauritius, Malasia, Mongolia, Mozambique, Morocco, Myanmar, Namibia, Niger, Nicaragua, Nigeria, Pakistan, Paraguay, Panama, Peru, Philippines, Rwanda, Senegal, South Africa, Sri Lanka, Tanzania, Tajikistan, Thailand, Togo, Turkmestan, Tunisia, Uganda, Uruguay, Viet Nam, Zambia and Zimbabwe.

2 Data for life expectancy is taken from https://ourworldindata.org/.

## References

[1] Ilo O Ilo S Simatele M. Climate change and health in the global south: a scientometric analysis. Dev South Afr. (2025) 42:708–27. doi: 10.1080/0376835X.2025.2588139

[2] Stott PA Christidis N Otto FEL Sun Y Vanderlinden J van Oldenborgh GJ . Attribution of extreme weather and climate-related events. WIREs Clim Change. (2016) 7:23–41. doi: 10.1002/wcc.380, 26877771 PMC4739554

[3] Ballester J Quijal-Zamorano M Méndez Turrubiates RF Pegenaute F Herrmann FR Robine JM . Heat-related mortality in Europe during the summer of 2022. Nat Med. (2023) 29:1857–66. doi: 10.1038/s41591-023-02419-z, 37429922 PMC10353926

[4] Cheung WWL Maire E Oyinlola MA Robinson JPW Graham NAJ Lam VWY . Climate change exacerbates nutrient disparities from seafood. Nat Clim Chang. (2023) 13:1242–9. doi: 10.1038/s41558-023-01822-1, 37927330 PMC10624626

[5] Braithwaite J Leask E Smith CL Dammery G Brooke-Cowden K Carrigan A . Analysing health system capacity and preparedness for climate change. Nat Clim Chang. (2024) 14:536–46. doi: 10.1038/s41558-024-01994-4

[6] Babugura A. Climate change and the global south: the case of Africa. Cairo: The Cairo Review of Global Affairs, The American University in Cairo (2021).

[7] Salameh AA Amin S Danish MH Asghar N Naveed RT Munir M. Socio-economic determinants of subjective wellbeing toward sustainable development goals: an insight from a developing country. Front Psychol. (2022) 13:13. doi: 10.3389/fpsyg.2022.961400, 36186294 PMC9515650

[8] Abdollahi E Champredon D Langley JM Galvani AP Moghadas SM. Temporal estimates of case-fatality rate for COVID-19 outbreaks in Canada and the United States. Can Med Assoc J. (2020) 192:E666–70. doi: 10.1503/cmaj.200711, 32444481 PMC7828851

[9] Pan L Biru A Lettu S. Energy poverty and public health: global evidence. Energy Econ. (2021) 101:105423. doi: 10.1016/j.eneco.2021.105423

[10] Frey BS Stutzer A. Happiness: A New Approach in Economics, CESifo DICE Report. (2010).

[11] Yan L Tao T Ullah S. Internet financial services, environmental technology, and happiness: implications for sustainable development. PLoS One. (2023) 18:e0287673. doi: 10.1371/journal.pone.0287673, 37939145 PMC10631691

[12] Doku I Phiri A. Climate finance and women-hunger alleviation in the global south: is the sub-Saharan Africa case any different? PLoS One. (2024) 19:e0290274. doi: 10.1371/journal.pone.0290274, 38315646 PMC10843080

[13] Bonasia M De Simone E D’Uva M Napolitano O. Environmental protection and happiness: a long-run relationship in Europe. Environ Impact Assess Rev. (2022) 93:106704. doi: 10.1016/j.eiar.2021.106704

[14] Aleksandrova M Kuhl L Malerba D. Unlocking climate finance for social protection: an analysis of the green climate fund. Clim Pol. (2024) 24:878–93. doi: 10.1080/14693062.2024.2338817

[15] MacGregor S Arora-Jonsson S Maeve Cohen M (2022). Caring in a Changing Climate: Centering care work in Climate action. Oxfam Research Backgrounder series. Available online at: https://www.oxfamamerica.org/explore/research-publications/caring-in-a-changing-climate/ (Accessed November 20, 2025).

[16] Liu Z Paan C. Newly evidence across the world on how climate financing helps in ensuring a greener future. Heliyon. (2024) 10:e34779. doi: 10.1016/j.heliyon.2024.e34779, 39148978 PMC11324983

[17] Gong X Borojo DG Yushi J. The heterogeneous impacts of climate finance on environmental sustainability and social welfare in developing countries. Kybernetes. (2025) 54:1904–37. doi: 10.1108/K-05-2023-0839

[18] Zoungrana TD Lompo AAB Toé DL. Effect of climate finance on environmental quality: a global analysis. Res Econ. (2024) 78:100989. doi: 10.1016/j.rie.2024.100989

[19] Yardımcı P Oskay C. The impact of green finance and financial globalization on environmental sustainability: empirical evidence from Türkiye. Sustainability. (2025) 17:5696. doi: 10.3390/su17135696

[20] Kelly AM. Battling for food security in Africa: is climate finance the missing bullet? World Food Policy. (2024) 10:227–53. doi: 10.1002/wfp2.12078

[21] Maddison D Rehdanz K Welsch H. Handbook on Wellbeing, Happiness and the Environment. London: Edward Elgar Publishing (2020).

[22] Lee C-C Li X Yu C-H Zhao J. The contribution of climate finance toward environmental sustainability: new global evidence. Energy Econ. (2022) 111:106072. doi: 10.1016/j.eneco.2022.106072

[23] Fang Y Lee C-C Li X. Financing a sustainable future: the effectiveness of climate finance across the primary, energy, and water sectors. Humanit Soc Sci Commun. (2025) 12:921. doi: 10.1057/s41599-025-04939-0

[24] Liu Y Dong K Nepal R. How does climate vulnerability affect the just allocation of climate aid funds? Int Rev Econ Financ. (2024) 93:298–317. doi: 10.1016/j.iref.2024.03.036

[25] Borghi J Garcia-Dorado SC Anton B Gerardo D Gasparri G Hanson M . Climate finance opportunities for health and health systems. Bull World Health Organ. (2024) 102:330–5. doi: 10.2471/BLT.23.290785, 38680468 PMC11046152

[26] Prasad R Singhania M. Impact of climate vulnerability on the allocation of climate finance: global evidence. J Environ Manag. (2026) 397:128307. doi: 10.1016/j.jenvman.2025.128307, 41401613

[27] Man Y. Psychological drivers of sustainability: examining happiness and progress toward the SDGs. Clean Prod Lett. (2025) 9:100103. doi: 10.1016/j.clpl.2025.100103

[28] Li W Wu D. Sustainability through business model innovation and climate finance in developing countries. Humanit Soc Sci Commun. (2025) 12:66. doi: 10.1057/s41599-024-04297-3

[29] Calvet L Gianfrate G Uppal R. The finance of climate change. J Corp Finan. (2022) 73:102162. doi: 10.1016/j.jcorpfin.2022.102162

[30] Ulfah IF Sukmana R Laila N Sulaeman S. A structured literature review on green sukuk (Islamic bonds): implications for government policy and future studies. J Islam Account Bus Res. (2024) 15:1118–33. doi: 10.1108/JIABR-10-2022-0255

[31] United Nations. Report of the World Commission on Environment and Development: Our Common Future. Oxford: Oxford University Press. (1987).

[32] Drexhage J Murphy D. Sustainable Development: from Brundtland to Rio 2012. New York, NY: Springerd (2010). p. 9–13.

[33] Barbier E. The concept of sustainable economic development, the economics of environment and development. London: Edward Elgar Publishing (1998).

[34] Sadiq M Ngo TQ Pantamee AA Khudoykulov K Thi Ngan T Tan LP. The role of environmental social and governance in achieving sustainable development goals: evidence from ASEAN countries. Econ Res Ekon Istraz. (2023) 36:170–90. doi: 10.1080/1331677X.2022.2072357

[35] Sachs JD. From millennium development goals to sustainable development goals. Lancet. (2010) 379:2206–11. 10.1016/S0140-6736(12)60685-0 22682467

[36] Lemke C. Conceptual framework of sustainable development. Cham: Springer (2021). p. 9–39.

[37] Robeyns I. The capability approach: a theoretical survey. J Hum Dev. (2005) 6:93–117. doi: 10.1080/146498805200034266

[38] Bigger P Webber S. Green structural adjustment in the World Bank’s resilient city. Ann Am Assoc Geogr. (2021) 111:36–51. doi: 10.1080/24694452.2020.1749023

[39] Doku I Richardson TE Essah NK. Bilateral climate finance and food security in developing countries: a look at German donations to sub-Saharan Africa. Food Energy Secur. (2022) 11:412. doi: 10.1002/fes3.412

[40] Kelly AM. Does climate finance foster happiness in African economies? Assessing the direct and indirect pathways. Glob Transit. (2025) 7:310–22. doi: 10.1016/j.glt.2025.05.003

[41] Zhao C Dong K Nepal R. Can climate finance effectively address the welfare losses arising from energy poverty? Energy Policy. (2026) 208:114655. doi: 10.1016/j.enpol.2025.114655

[42] Kabutey M Nakouwo SN Taden J. How does climate finance affect the ease of doing business in recipient countries? J Risk Financ Manag. (2025) 18:263. doi: 10.3390/jrfm18050263

[43] Donath L Oprea I. The inclusive finance factor of happiness. J Public Adm Finance Law. (2020) 18:139–49.

[44] Tran MQ Luu HN Luu NH Nguyen MD Phan NTD. Impact of climate-related financial policy on sustainable development. J Environ Assess Policy Manag. (2024) 26:27. doi: 10.1142/S1464333224400027

[45] Weiler F Klöck C Dornan M. Vulnerability, good governance, or donor interests? The allocation of aid for climate change adaptation. World Dev. (2018) 104:65–77. doi: 10.1016/j.worlddev.2017.11.001

[46] Zhang Z Zhao M Zhang Y Feng Y. How does urbanization affect public health? New evidence from 175 countries worldwide. Front Public Health. (2023) 10:964. doi: 10.3389/fpubh.2022.1096964, 36684862 PMC9852986

[47] Aburto JM Schöley J Kashnitsky I Zhang L Rahal C Missov TI . Quantifying impacts of the COVID-19 pandemic through life-expectancy losses: a population-level study of 29 countries. Int J Epidemiol. (2022) 51:63–74. doi: 10.1093/ije/dyab207, 34564730 PMC8500096

[48] Pavot W Diener E. The satisfaction with life scale and the emerging construct of life satisfaction. J Posit Psychol. (2008) 3:137–52. doi: 10.1080/17439760701756946

[49] Ko J Leung CK Chen X Palmer DA. From emissions to emotions: exploring the impact of climate change on happiness across 140 countries. Glob Transit. (2024) 6:231–40. doi: 10.1016/j.glt.2024.10.005

[50] Ionescu-Feleagă L Ionescu B-Ș Stoica OC. The impact of digitalization on happiness: a European perspective. Mathematics. (2022) 10:2766. doi: 10.3390/math10152766

[51] Hadipour A Delavari S Bayati M. What is the role of institutional quality in health outcomes? A panel data analysis on 158 countries from 2001-2020. Heliyon. (2023) 9:e20251. doi: 10.1016/j.heliyon.2023.e20251, 37809989 PMC10560016

[52] Njangang H Padhan H Tiwari AK. From aid to resilience: assessing the impact of climate finance on energy vulnerability in developing countries. Energy Econ. (2024) 134:107595. doi: 10.1016/j.eneco.2024.107595

[53] Baum CF Schaffer ME Stillman S. Instrumental variables and GMM: estimation and testing. Stata J Promoting Commun Stat Stata. (2003) 3:1–31. doi: 10.1177/1536867X0300300101

[54] Kushawaha D. <article-title update="added">understanding the role of greenfield and mergers & acquisitions foreign direct investments in renewable energy expansion in developing countries. Energy Econ. (2025) 145:108450. doi: 10.1016/j.eneco.2025.108450

[55] Acheampong AO Dzator J Shahbaz M. Empowering the powerless: does access to energy improve income inequality? Energy Econ. (2021) 99:105288. doi: 10.1016/j.eneco.2021.105288

[56] Zhao C Dong K Lee C-C. Carbon lock-in endgame: can energy trilemma eradication contribute to decarbonization? Energy. (2024) 293:130662. doi: 10.1016/j.energy.2024.130662

[57] Dabbous A Aoun Barakat K Ben Arfi W Nammouri H. The impact of environmental policy stringency and economic complexity on nations’ energy transitions: the mediating role of fintech financing. Energy Econ. (2025) 147:108540. doi: 10.1016/j.eneco.2025.108540

[58] Scheelbeek PFD Dangour AD Jarmul S Turner G Sietsma AJ Minx JC . The effects on public health of climate change adaptation responses: a systematic review of evidence from low- and middle-income countries. Environ Res Lett. (2021) 16:073001. doi: 10.1088/1748-9326/ac092c, 34267795 PMC8276060

[59] Hogan MJ Leyden KM Conway R Goldberg A Walsh D McKenna-Plumley PE. Happiness and health across the lifespan in five major cities: the impact of place and government performance. Soc Sci Med. (2016) 162:168–76. doi: 10.1016/j.socscimed.2016.06.030, 27359322

[60] Azam I Guo Y Naseer MM. The sustainability challenge and social benefits of industrialisation in NICs. Resour Policy. (2025) 103:105541. doi: 10.1016/j.resourpol.2025.105541

[61] Dickin S Bayoumi M Giné R Andersson K Jiménez A. Sustainable sanitation and gaps in global climate policy and financing. NPJ Clean Water. (2020) 3:24. doi: 10.1038/s41545-020-0072-8

[62] Khan M Robinson S Weikmans R Ciplet D Roberts JT. Twenty-five years of adaptation finance through a climate justice lens. Clim Chang. (2020) 161:251–69. doi: 10.1007/s10584-019-02563-x

[63] Trinh HH. Climate finance, policy uncertainty, and global financial stability: a systematic review through bibliometric analysis. Cham: Springer (2024). p. 209–25.

[64] Krekel C MacKerron G. How environmental quality affects our happiness. World Happiness Rep. (2020) 95:112

[65] Machado JAF Santos Silva JMC. Quantiles via moments. J Econom. (2019) 213:145–73. doi: 10.1016/j.jeconom.2019.04.009

[66] Shobande OA Ogbeifun L. Pooling cross-sectional and time series data for estimating causality between technological innovation, affluence and carbon dynamics: a comparative evidence from developed and developing countries. Technol Forecast Soc Change. (2023) 187:122192. doi: 10.1016/j.techfore.2022.122192

[67] Yitayaw MK Mogess YK Feyisa HL Mamo WB Abdulahi SM. Determinants of bank stability in Ethiopia: a two-step system GMM estimation. Cogent Econ Financ. (2023) 11:1771. doi: 10.1080/23322039.2022.2161771

[68] Danish M Khan H. Social capital as a resource of subjective wellbeing: mediating role of health status. Forman J Econ Stu. (2020) 16:1608. doi: 10.32368/FJES.20201608

[69] Nayna Schwerdtle P Ngo TA Hasch F Phan TV Quitmann C Montenegro-Quiñonez CA. Climate change resilient health facilities: a scoping review of case studies in low and middle-income countries. Environ Res Lett. (2024) 19:074041. doi: 10.1088/1748-9326/ad472b

