# Supplementary material for: The impacts of development finance for climate on public health outcomes and life satisfaction: evidence from developing countries in the global south
Source: Front Public Health. 2026 Apr 29;14:1775273. doi: 10.3389/fpubh.2026.1775273 (PMC13167978; doi:10.3389/fpubh.2026.1775273)
Supplement: Supplementary file 1 [file Supplementary_file_1.pdf]

Supplementary Material

Table S1. Multicollinearity test results.

**Panel I:** DFC and crude death rate specification		
Variables	IVF	1/IVF
$digital_{it}$	2.69	0.372
$urban_{it}$	2.04	0.491
IQ_{it}	1.8	0.555
$industry_{it}$	1.8	0.555
$health_{it}$	1.73	0.578
$trade_{it}$	1.38	0.722
FDI_{it}	1.17	0.854
DFC_{it}	1.15	0.873
$growth_{it}$	1.13	0.884
Mean IVF	1.65	
**Panel II**: DFC and life satisfaction specification		
Variables	IVF	1/IVF
$digital_{it}$	2.76	0.363
$urban_{it}$	2.06	0.486
IQ_{it}	1.82	0.551
$industry_{it}$	1.72	0.582
$health_{it}$	1.72	0.583
$trade_{it}$	1.38	0.725
FDI_{it}	1.18	0.850
DFC_{it}	1.15	0.869
$growth_{it}$	1.14	0.881
Mean IV	1.66	

Table S2. The effects of DFC on crude death rate and life satisfaction (FGLS)

**Variables**	**(I)**<br>(Crude death rate)	**(II)**<br>(Life satisfaction
DFC_{it}	-.0170***	0.025***
	(0.002)	(0.008)
Control variables	Yes	Yes
Time FE	Yes	Yes
Obs.	731	651
Groups	69	69
Wald chis2(19)	337.21***	1569.20***

Notes: ***<0.01

Table S3. The effects of DFC on crude death rate and life satisfaction (MLE)

Variables	(I)<br>(Crude death rate)	(II)<br>(Life satisfaction)
DFC_{it}	-0.003***<br>(0.002)	0.032***<br>(0.010)
Control variables	Yes	Yes
Time FE	Yes	Yes
Obs.	731	723
Log likelihood	664.567	-344.5802
Wald chis2(19)	272.74***	102.06***

Notes: ***<0.01

Table S4. The effects of DFC on crude death rate and life satisfaction (two-step system GMM).

Variables	(I)<br>(Crude death rate)	(II)<br>(Life satisfaction)
DFC_{it}	-0.016***<br>(0.002)	0.026**<br>(0.012)
Control variables	Yes	Yes
Time FE	Yes	Yes
Obs.	731	651
Groups	69	69
No. of instruments	59	63
Wald chi2(19)	68812.57***	45015.66***
AB2	0.151	0.220
Hansen (p-value)	0.101	0.432

Notes: **<0.05, ***<0.01

Table S5. The mediation role of crude death rate (GSEM).

Variables	(I)<br>(Crude death rate)	(II)<br>Life satisfaction
$Crudedeath_{it}$		-0.429***<br>(0.920)
DFC_{it}	-0.022***<br>(0.005)	0.034***<br>(0.012)
Controls	Yes	Yes
Time FE	Yes	Yes
Obs.		660
Log-likelihood		-612.696

Notes: ***<0.01

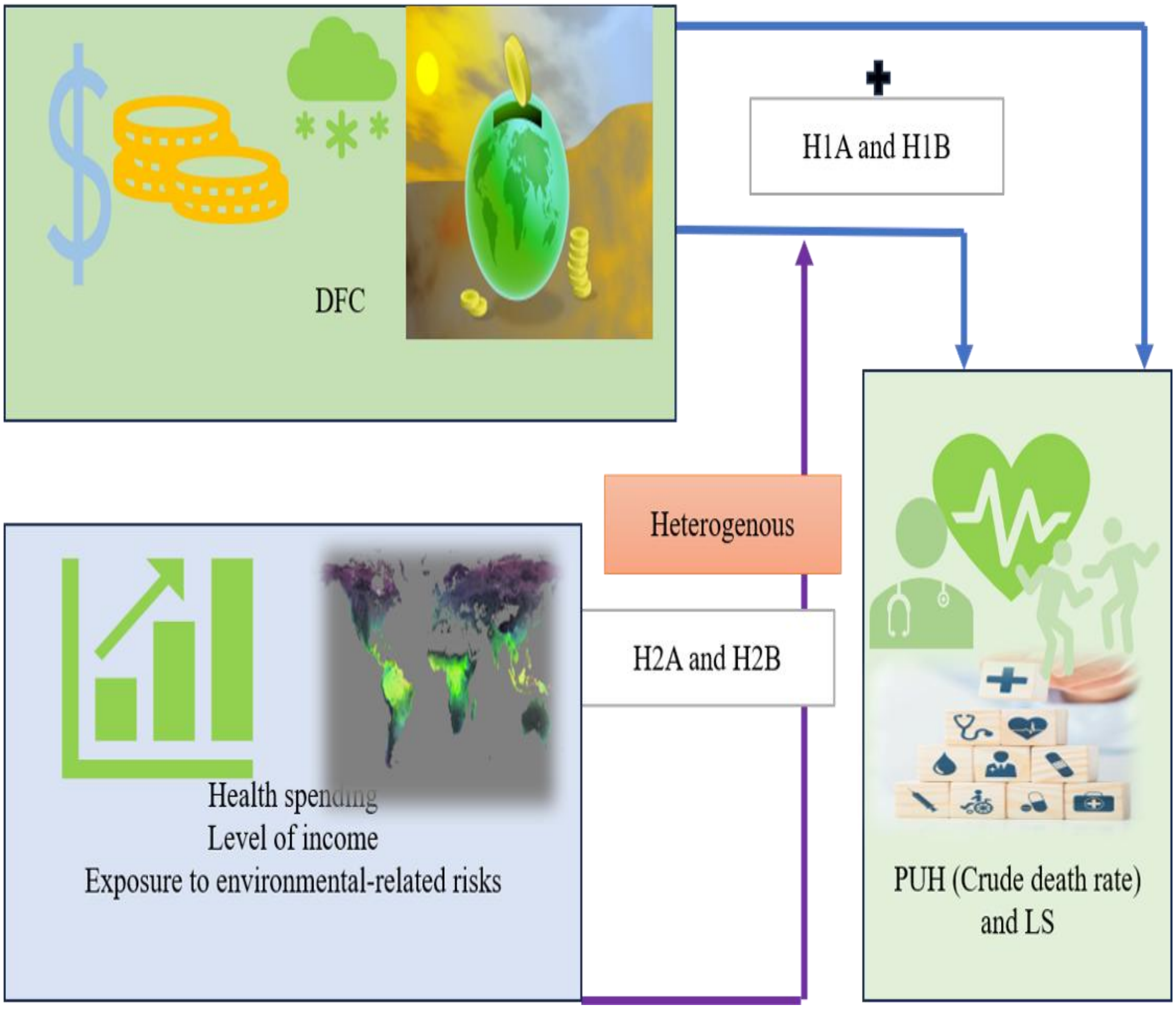

Figure S1

Hypotheses framework.

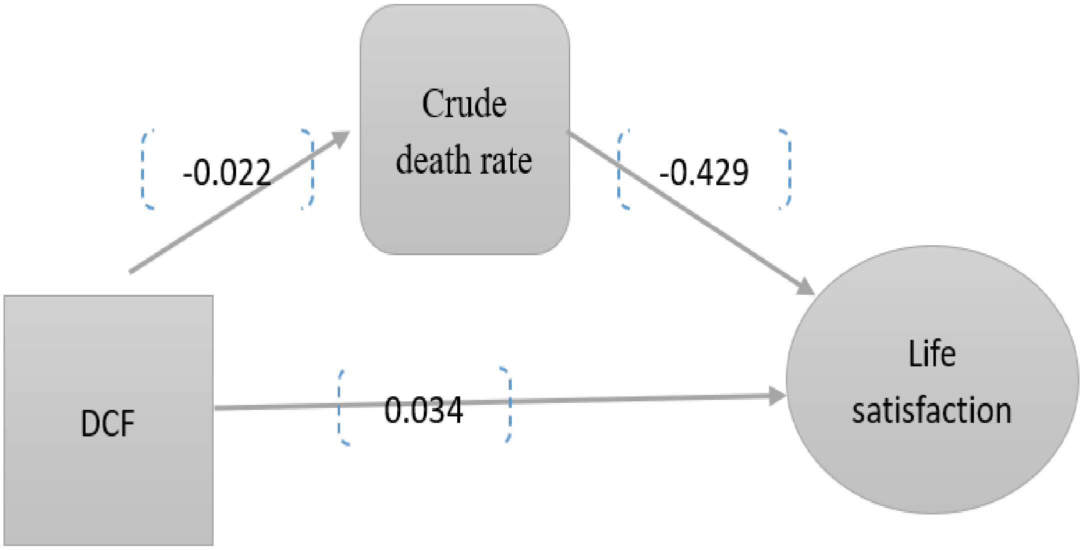

Figure S2

Mediation role of crude death rate.
